# Long-Range Detection of a Wirelessly Powered Resistive Transducer

**DOI:** 10.1109/tim.2022.3178475

**Published:** 2022-05-27

**Authors:** Wei Qian, Chunqi Qian

**Affiliations:** Department of Electrical and Computer Engineering, Michigan State University, East Lansing, MI 48824 USA.; Department of Radiology, Michigan State University, East Lansing, MI 48824 USA

**Keywords:** Frequency modulation, nonlinear circuits, oscillators, resistive sensors, wireless power transmission

## Abstract

Wireless measurement of resistance variation is particularly desirable inside confined cavities where wire connection and battery replacement are undesirable. Compared to capacitive or inductive transducers, resistive transducers have better availability, whose resistance changes can be directly converted into detectable voltages by electric bridges. However, to wirelessly operate electric bridges on batteryless platforms, multistage circuits are required to convert dc signals into wireless signals, making the whole system hard to miniaturize without using complicated integrated circuits. Alternatively, resistive transducers can be incorporated into passive LC resonators for contactless characterization by the backscattering method. This design, however, is ineffective beyond the near field, and it requires complicated line shape analysis of resonators’ frequency response curves. Here, we will significantly improve the remote detectability of a resistive transducer, by inductively coupling it with a parametric resonator. Upon activation by wireless pumping power with an external antenna, the parametric resonator can self-oscillate and emit strong oscillation signals. The temperature-induced resistance change is converted into linear frequency shifts of the oscillation signal that can be detected over large distance separations for up to 20-fold the sensor’s own dimension. Every 0.1 °C of temperature change can be converted into 8 kHz of frequency shift that is approximately threefold larger than the linewidth of oscillation peak. This sensor maintains good linearity between 25 °C and 41 °C, providing enough range for physiological monitoring. In conclusion, we have fabricated a resistance-to-frequency converter for remote detection of resistance changes via a wirelessly powered parametric oscillator. Besides this proof-of-concept demonstration for temperature sensing, the general concept of resistance-to-frequency conversion will improve the remote detectability of a broad range of resistive transducers for physiological and environmental monitoring.

## I. Introduction

RESISTIVE transducers [1]–[4] are widely utilized to convert environmental parameters (e.g., temperature and pressure) into electrical signals. Compared to capacitive [5]–[8] or inductive [9]–[12] transducers, resistive transducers are often easier to fabricate and more readily available. To quantify resistance changes on a sensing platform, a common method is to compare voltage drops between the sensing resistor and a reference resistor in an electric bridge [13]. This design, however, is most suitable for circuits with wired connections. To wirelessly transmit sensing information from the inside of confined cavities, voltage drops across the electric bridge can be encoded onto a wireless carrier wave as analog sidebands by a voltage-to-frequency converter [14] or as digital waveforms [15], [16] by an A/D converter. These approaches can transmit information about resistance variation over a distance separation of at least ninefold the sensor’s dimension. However, because these approaches rely on dc power to operate, additional circuit modules, such as rectifiers and voltage regulators, are required to convert RF power into dc power [17]–[22]. Even though the voltage regulator, the RF transmitter, and the temperature sensor can in principle be integrated into a single chip [23]–[25], commercial off-the-shelf integrated circuits are normally enclosed inside centimeter-scale packages. Such an IC chip is hard to fit inside confined body cavities, not to mention the additional power harvesting antenna and voltage biasing circuitry that are required to operate the IC [26]–[28]

Alternatively, passive LC resonators have been utilized to estimate resistance changes. By connecting the sensing resistor in parallel [29]–[32] or in series [33], [34] to an LC resonator, resistance changes can affect the line shape of the resonator’s frequency response curve that can be inductively measured by an external network analyzer. However, line shape analysis is a complex nonlinear procedure, leading to lower temperature resolution (1.2 °C in Table I). Moreover, direct measurement of the frequency response curve is effective only when the detection antenna is close enough to the resonator [35]. When the distance separation is larger than the passive resonator’s own dimension, backscattered signals from the resonator will be much smaller than the environmental background, making the frequency response curve completely buried beneath the instrumental noise floor. Although the resonance behavior of a remote passive sensor can be interrogated after stimulation with multifrequency excitation pulses [36]–[40], specialized read-out apparatuses are required to separate frequency-specific backscattered signals in the frequency or time domain, making the signal decoding process complex and expensive to implement.

In this work, we are going to greatly improve the remote detectability of a wireless resistive sensor by inducing self-oscillation currents inside the resonator, making the circuit’s oscillation frequency linearly modulated by resistance change. More specifically, we will replace the constant-valued capacitors in an ordinary LC resonator with two variable capacitors that are symmetrically connected in the head-to-head configuration. By bridging the virtual voltage grounds across this parametric resonator, a second resonance mode is created to facilitate multiband energy exchange. Upon activation by a pumping field provided at the sum frequency of its two lower resonance modes, the resonator can utilize wireless pumping power to sustain circuit oscillation defined by its two lower resonance modes. By overlaying this double-frequency parametric resonator with a loop inductor that is serially connected with a resistive transducer, resistance change in the coupled loop can be converted into reactance change in the parametric resonator so that the resultant resonance frequency shift can be remotely detected as linear frequency shift of the oscillation signal. Compared to passive sensors that can only be characterized by the backscattering method in the near field, this frequency-encoded oscillator is easier to detect, even when it is separated from the detection antenna by 20-fold its own dimension. Without the need for rectifiers, voltage regulators, or RF transmitters, this compact and wirelessly powered resistive sensor will become useful to monitor a broad range of physiological or environmental parameters that can induce resistance changes from the inside of confined cavities.

## II. Operation Principle

### A. Resistance-Dependent Resonance Frequency Shift

**Fig. 1.**
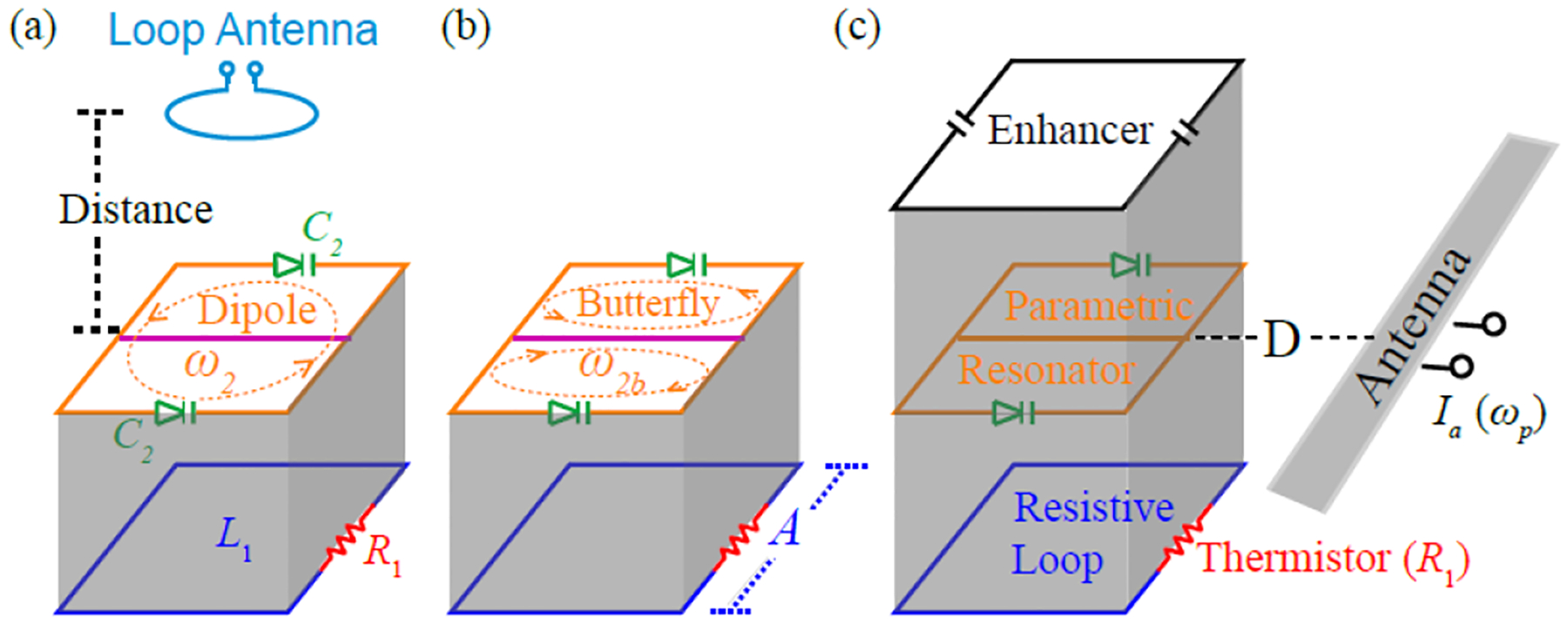
(a) Sensor consists of a parametric resonator (orange) that is inductively coupled to a resistive loop (blue) so that resistance change in the thermistor (red) is converted into linear shift of resonance frequency. This frequency shift can in principle be measured by a loop antenna (cyan) using passive backscattering. (b) To improve the sensor’s remote detectability, an additional conductor (pink) bridges the virtual voltage grounds of the parametric resonator, creating a second resonance mode to facilitate multiband energy exchange via nonlinear capacitors (green), enabling sustained circuit oscillation upon activation by wireless pumping power. (c) To improve the efficiency of pumping power, the double-frequency resonator is overlaid by another single-frequency resonant enhancer that can locally concentrate magnetic flux at the sum frequency of the dipole and the butterfly modes.

As shown in Fig. 1(a), the core components of the wireless sensor include a resistive loop (blue) coaxially overlaid with a parametric resonator (orange). Through inductive coupling, resistance change in the former is converted into reactance change in the latter. The effective impedance inside the parametric resonator is

(1) $$\text{Z}=\frac{\omega^{2}M^{2}\left(R_{1}-j\omega L_{1}\right)}{R_{1}^{2}+\omega^{2}L_{1}^{2}}+R_{2}+j\omega L_{2}+\frac{2}{j\omega C_{2}}\;=\frac{\omega^{2}M^{2}}{R_{1}+\omega^{2}L_{1}^{2}/R_{1}}+R_{2}+j\;\left(-\frac{\omega^{2}M^{2}\omega L_{1}}{R_{1}^{2}+\omega^{2}L_{1}^{2}}+\omega L_{2}-\frac{2}{\omega C_{2}}\right)\;\equiv R_{2d}+jX_{2d}$$

where $R_{1}$ and $L_{1}$ are the sensing resistance and the inductance in the resistive loop, respectively, $R_{2d}$ and $L_{2d}$ are the effective resistance and inductance of the parametric resonator (in its dipole mode), respectively, and $C_{2}$ is the effective capacitance of each varactor under zero voltage bias. The resonance frequency $\omega_{2}$ can be estimated by making the imaginary part of (1) equal to zero

(2) $$\omega_{2}^{2}=\frac{L_{1}^{2}-C_{2}L_{2}R_{1}^{2}/2}{C_{2}L_{1}\left(L_{1}L_{2}-M^{2}\right)}\;+\frac{\sqrt{{\left(2L_{1}^{2}+C_{2}L_{2}R_{1}^{2}\right)}^{2}-8C_{2}R_{1}^{2}L_{1}M^{2}}}{2C_{2}L_{1}\left(L_{1}L_{2}-M^{2}\right)}.$$

By correlating $C_{2}$ with the self-resonance frequency $\omega_{20}$ of the parametric resonator in its stand-alone configuration, i.e., $C_{2}=2/\left(\omega_{20}^{2}L_{2}\right)$, (2) becomes

(3) $$\omega_{2}^{2}=\frac{\omega_{20}^{2}\left(1-\frac{R_{1}^{2}}{\omega_{20}^{2}L_{1}^{2}}\right)}{2\left(1-M^{2}/\left(L_{1}L_{2}\right)\right)}\;+\frac{\omega_{20}^{2}\sqrt{{\left(1+\frac{R_{1}^{2}}{\omega_{20}^{2}L_{1}^{2}}\right)}^{2}-\left(\frac{4R_{1}^{2}}{\omega_{20}^{2}L_{1}^{2}}\right)\left(\frac{M^{2}}{L_{1}L_{2}}\right)}}{2\left(1-M^{2}/\left(L_{1}L_{2}\right)\right)}\;=\frac{\omega_{20}^{2}\left(1-\frac{R_{1}^{2}}{\omega_{20}^{2}L_{1}^{2}}\right)}{2\left(1-\kappa^{2}\right)}\;+\frac{\omega_{20}^{2}\left(1+\frac{R_{1}^{2}}{\omega_{0}^{2}L_{1}^{2}}\right)\sqrt{1-\kappa^{2}\left(\frac{4R_{1}^{2}}{\omega_{20}^{2}L_{1}^{2}}\right)/{\left(1+\frac{R_{1}^{2}}{\omega_{20}^{2}L_{1}^{2}}\right)}^{2}}}{2\left(1-\kappa^{2}\right)}$$

where $\kappa^{2}=M^{2}/\left(L_{1}L_{2}\right)$ is the coupling coefficient that is determined by geometric proximity between the parametric resonator and the resistive loop. Because the mutual inductance is normally much smaller than the self-inductance (i.e., $\kappa^{2}\ll 1$), $4\kappa^{2}R_{1}^{2}/\left(\omega_{20}^{2}L_{1}^{2}\right)\ll 1$ also holds true. Equation (3) can thus be approximated as

(4) $$\omega_{2}^{2}\approx \frac{\omega_{20}^{2}\left(1-\frac{R_{1}^{2}}{\omega_{20}^{2}L_{1}^{2}}\right)}{2\left(1-k^{2}\right)}\;+\frac{\omega_{20}^{2}\left(1+\frac{R_{1}^{2}}{\omega_{20}^{2}L_{1}^{2}}\right)\left(1-\left(\frac{2\kappa^{2}R_{1}^{2}}{\omega_{20}^{2}L_{1}^{2}}\right)/{\left(1+\frac{R_{1}^{2}}{\omega_{20}^{2}L_{1}^{2}}\right)}^{2}\right)}{2\left(1-\kappa^{2}\right)}\;=\frac{\omega_{20}^{2}}{\left(1-\kappa^{2}\right)}-\frac{\omega_{20}^{2}\kappa^{2}\frac{R_{1}^{2}}{\omega_{20}^{2}L_{1}^{2}}}{\left(1-\kappa^{2}\right)\left(1+\frac{R_{1}^{2}}{\omega_{20}^{2}L_{1}^{2}}\right)}\;=\omega_{20}^{2}+\frac{\omega_{20}^{2}\kappa^{2}}{\left(1-\kappa^{2}\right)}\frac{1}{\left(1+\frac{R_{1}^{2}}{\omega_{20}^{2}L_{1}^{2}}\right)}.$$

Therefore, compared to the parametric resonator in its standalone configuration, the resistive loop will change its resonance frequency by a factor of

(5) $$\frac{\omega_{2}}{\omega_{20}}\;={\left(1+\frac{\kappa^{2}}{\left(1+\frac{R_{1}^{2}}{\omega_{20}^{2}L_{1}^{2}}\right)\left(1-\kappa^{2}\right)}\right)}^{\frac{1}{2}}\approx 1+\frac{\kappa^{2}}{2\left(1+\frac{R_{1}^{2}}{\omega_{20}^{2}L_{1}^{2}}\right)\left(1-\kappa^{2}\right)}.$$

Normally, the relative change of the sensing resistance ($\text{d}R_{1}/R_{1}$) is proportional to the change in environmental parameter (e.g., temperature dT). We can thus establish the relation between sensing resistance and environmental parameter, by taking the derivative of (5) with respect to $\text{d}R_{1}/R_{1}$

(6) $$-\frac{d\left(\omega_{2}/\omega_{20}\right)}{dR_{1}/R_{1}}\approx \frac{\kappa^{2}}{\left(1-\kappa^{2}\right){\left(\frac{R_{1}}{L_{1}\omega_{20}}+\frac{L_{1}\omega_{20}}{R_{1}}\right)}^{2}}.$$

### B. Conversion to Oscillation Frequency Shift

Although the resistance-dependent frequency shift can in principle be measured from the frequency response curve of the coupled resonator, such measurement can be challenging when the resonance frequency shift is much smaller than the bandwidth of the frequency response curve or when the resonator is remotely coupled to the external detection antenna. To improve the measurement accuracy of small frequency shift over large distance separations, the coupled parametric resonator needs to be activated by wireless pumping power, producing a sharp oscillation peak whose frequency shift can be sensitively detected over larger distance separations.

**Fig. 2.**
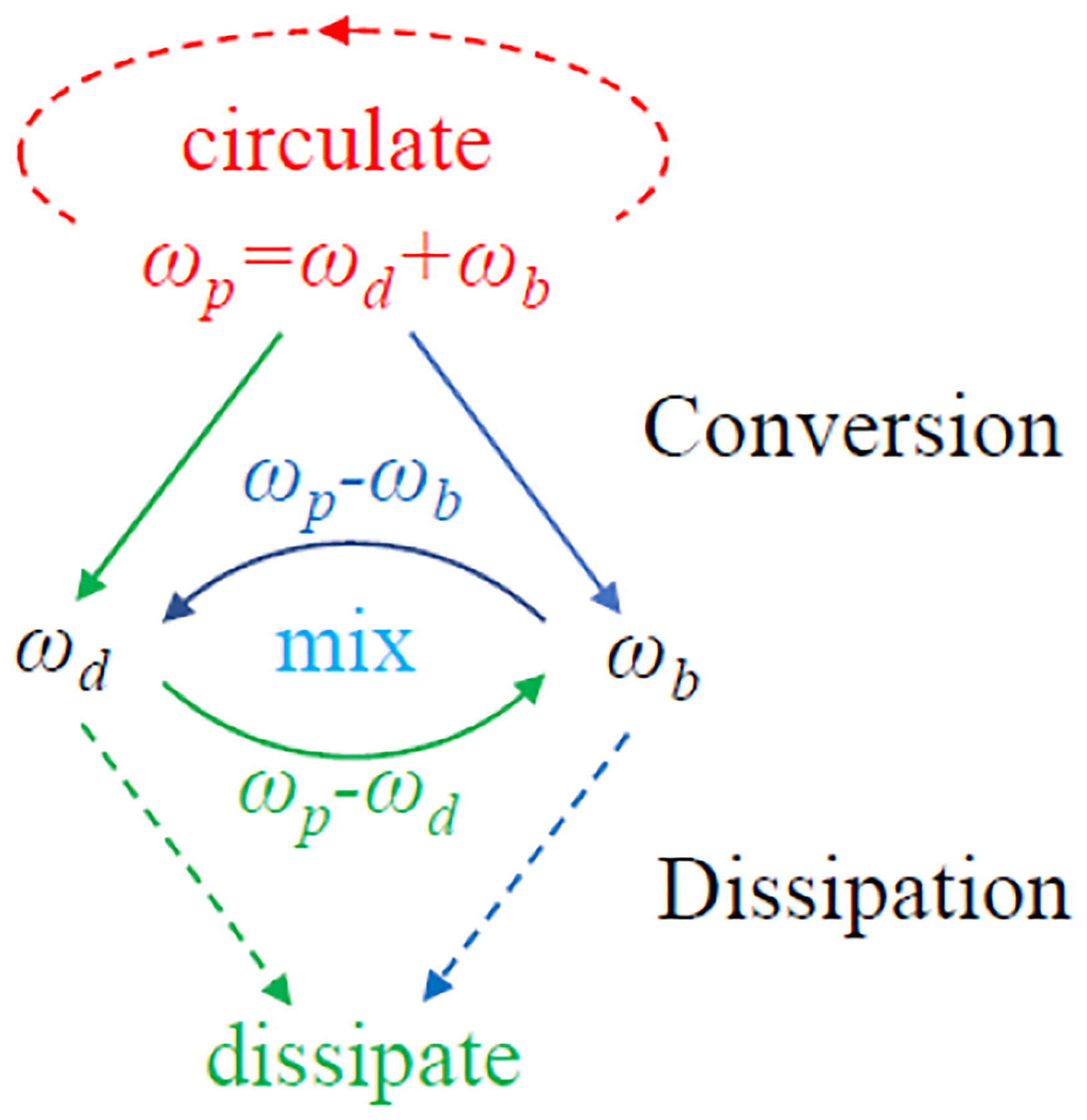
Operation principle of parametric oscillator. When the pumping frequency is equal to the sum of resonance frequencies, a residual signal at $\omega_{d}$ can interact with the pumping signal to generate an amplified signal at $\omega_{b}=\omega_{p}-\omega_{d}$. This amplified signal in the second resonance mode can again interact with the pumping signal to generate further enlarged signal in the first resonance mode, thus completing one energy exchange cycle. Under stable oscillation conditions, the fraction of energy converted from the pumping signal should be equal to the fraction of energy dissipated in the two resonance modes.

More specifically, the coupled resonator also has a second (butterfly) resonance mode at $\omega_{2b}$ [Fig. 1(b)] that is created by bridging the virtual grounds of its first (dipole) resonance mode. Although this butterfly mode does not directly interact with the resistive loop, it is necessary to sustain circuit oscillation through multiband frequency mixing. By adjusting the pumping frequency equal to the sum of the two resonance frequencies $\left(\omega_{p}=\omega_{2}+\omega_{2b}\right)$, energy exchange between resonance modes is facilitated by nonlinear capacitors [41], thus enhancing the oscillation currents inside both modes via positive feedback (Fig. 2).

When the dipole mode resonance frequency $\omega_{2}$ slightly shifts due to resistance variation in the coupling loop, $\omega_{2}+\omega_{2b}$ will be slightly deviated from the pumping frequency $\omega_{p}$, making the oscillation frequency of each mode slightly deviated from its resonance frequency. The frequency deviation can be estimated by making the reactance-to-resistance ratio equal for both the dipole and butterfly modes

(7) $$\frac{X_{\text{dipole}}}{R_{\text{dipole}}}=\frac{2\left(\omega_{d}-\omega_{2}\right)L_{2}}{R_{2d}}=\frac{X_{\text{butterfly}}}{R_{\text{butterfly}}}=\frac{2\left(\omega_{b}-\omega_{2b}\right)L_{2b}}{R_{2b}}$$

where $\omega_{d}$ and $\omega_{b}$ are oscillation frequencies that are slightly deviated from the dipole and butterfly mode resonance frequencies. $\omega_{2}$ is the dipole mode resonance frequency that is modulated by the thermistor, and $\omega_{2b}$ is the resonance frequency of the butterfly mode that is unaffected by resistance change. $L_{2}$ and $L_{2b}$ are effective inductance of the dipole and butterfly modes that are determined by circuit dimensions, $R_{2}$ and $R_{2b}$ are effective resistance of the dipole and butterfly modes. By plugging $\omega_{p}=\omega_{d}+\omega_{b}$ into (7), the oscillation frequency of the dipole mode can be expressed as

(8) $$\omega_{d}=\frac{\omega_{2}L_{2}/R_{2d}-\omega_{2b}L_{2b}/R_{2b}+\omega_{p}L_{2b}/R_{2b}}{L_{2b}/R_{2b}+L_{2}/R_{2d}}\;=\omega_{2}\left(1-\frac{L_{2b}/R_{2b}}{L_{2b}/R_{2b}+L_{2}/R_{2d}}\right)\;+\left(\omega_{p}-\omega_{2b}\right)\left(\frac{L_{2b}/R_{2b}}{L_{2b}/R_{2b}+L_{2}/R_{2d}}\right).$$

By taking the derivative of (8) with respect to $dR_{1}/R_{1}$

(9) $$\frac{d\left(\omega_{d}/\omega_{20}\right)}{dR_{1}/R_{1}}\;=\frac{d\left(\omega_{2}/\omega_{20}\right)}{dR_{1}/R_{1}}\left(1-\frac{L_{2b}/R_{2b}}{L_{2b}/R_{2b}+L_{2}/R_{2d}}\right)\;+\frac{\left(\omega_{p}-\omega_{2b}-\omega_{2}\right)}{\omega_{20}}R_{1}\frac{d}{dR_{1}}\left(\frac{L_{2b}/R_{2b}}{L_{2b}/R_{2b}+L_{2}/R_{2d}}\right).$$

The second line of (9) is negligible, which will be proved in the Appendix. As a result, (9) is left with the first line that describes the proportional relation between the relative change in oscillation frequency $d\left(\omega_{d}/\omega_{20}\right)$ and the relative change in resonance frequency $d\left(\omega_{2}/\omega_{20}\right)$. By plugging (6) into (9)

(10) $$-\frac{d\left(\omega_{d}/\omega_{20}\right)}{dR_{1}/R_{1}}\;\approx -\frac{d\left(\omega_{2}/\omega_{20}\right)}{dR_{1}/R_{1}}\left(1-\frac{L_{2b}/R_{2b}}{L_{2b}/R_{2b}+L_{2}/R_{2d}}\right)\;\approx \frac{\kappa^{2}}{\left(1-\kappa^{2}\right){\left(\frac{R_{1}}{L_{1}\omega_{20}}+\frac{L_{1}\omega_{20}}{R_{1}}\right)}^{2}}\left(1-\frac{L_{2b}/R_{2b}}{L_{2b}/R_{2b}+L_{2}/R_{2d}}\right)\;\leq \frac{\kappa^{2}}{4\left(1-\kappa^{2}\right)}\left(1-\frac{L_{2b}/R_{2b}}{L_{2b}/R_{2b}+L_{2}/R_{2d}}\right).$$

Equation (10) quantifies the ratio between the relative change in oscillation frequency $d\left(\omega_{d}/\omega_{20}\right)$ and the relative change in sensing resistance $dR_{1}/R_{1}$. This ratio will reach maximum magnitude when $R_{1}\sim L_{1}\omega_{20}$. When $R_{1}/\omega_{20}L_{1}$ changes from 0.8 to 1.2, (10) maintains 95% of its maximum value, leading to a good linear response. By plugging $\text{d}R_{1}/R_{1}=\beta \text{d}T$ into (10), we can establish the linear relation between the oscillation frequency and temperature

(11) $$-\frac{d\left(\omega_{d}/\omega_{20}\right)}{\beta dT}\leq \frac{\kappa^{2}}{4\left(1-\kappa^{2}\right)}\left(1-\frac{L_{2b}/R_{2b}}{L_{2b}/R_{2b}+L_{2}/R_{2d}}\right).$$

## III. Circuit Construction

**Fig. 3.**
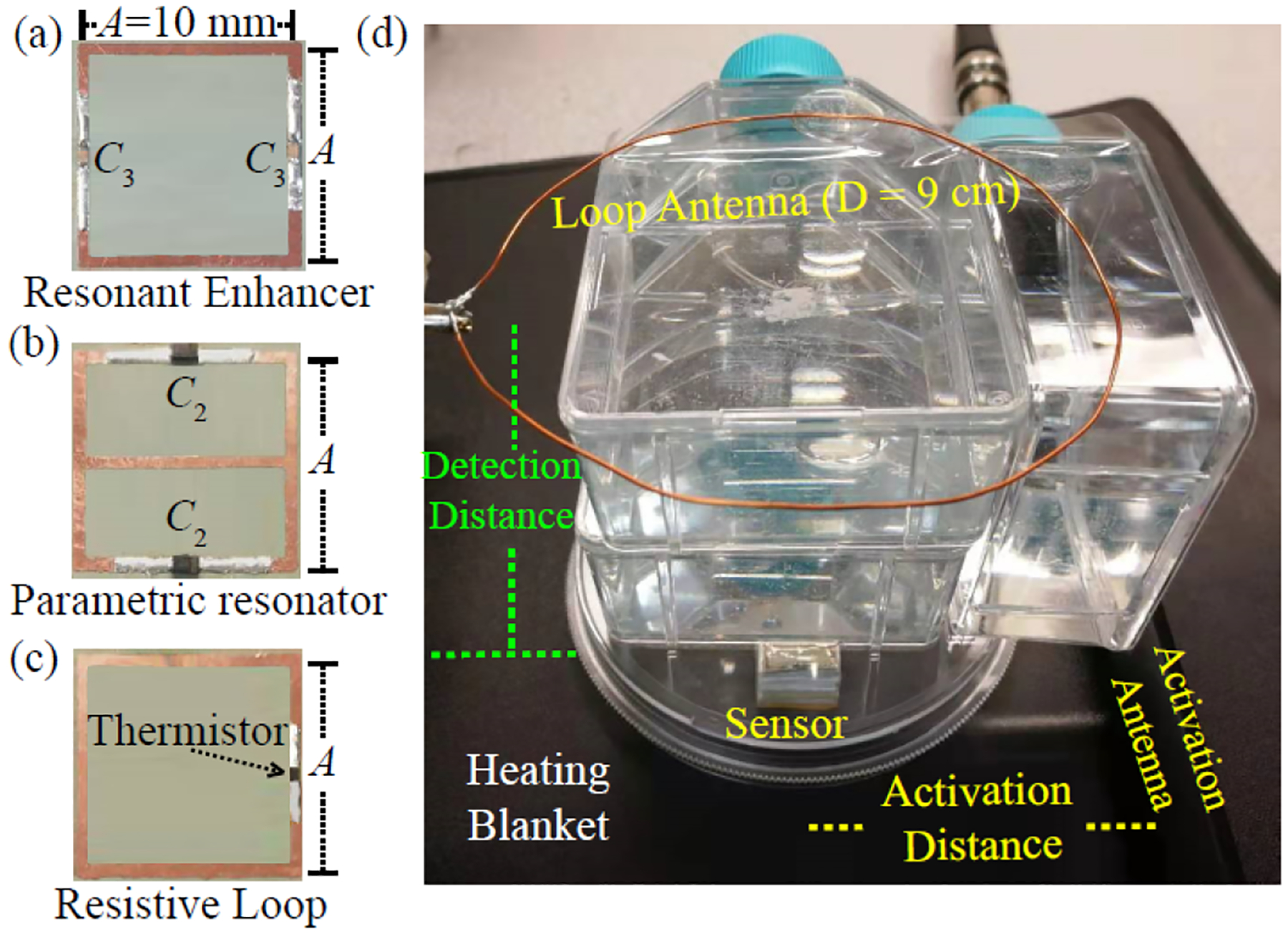
(a) Resonant enhancer for the pumping field was made by filling the gaps of a 10 × 10 mm^2^ conductor loop with two 2.7-pF chip capacitors. (b) Parametric resonator was made by filling the gaps of its top and bottom conductors with two varactors (BBY53-02W). (c) Resistive loop was made by filling the single gap of a 10 × 10 mm^2^ loop with a thermistor (ERT-J1VA101H) whose resistance at ambient temperature was approximately equal to the reactive impedance of the square conductor at $\omega_{20}=471.6\;\text{MHz}$ (the dipole mode resonance frequency of the parametric resonator in its standalone configuration). (d) After overlaying the circuits in (a)–(c) together, the sensor was placed on a heating blanket for temperature control. When the sensor was wirelessly activated by a 17.6-cm activation antenna (black rod), its temperature-modulated oscillation signal could be remotely detected by a 9-cm loop antenna (orange loop) placed above the sensor and connected to a spectrum analyzer. The gap between the sensor and the detection (or activation) antenna was varied up to 20 cm by changing the number of flasks lying against each other. Each flask contains a solution of 146% sucrose and 3.6% NaCl to emulate the effect of dissipative tissues.

The wireless resistive sensor consisted of a resonant enhancer [Fig. 3(a)], a parametric resonator [Fig. 3(b)], and a resistive loop [Fig. 3(c)]. The resistive loop [Fig. 3(c)] was fabricated by etching a square conductor pattern on a copper-clad polyimide film. For prototype demonstration, the conductor pattern had a dimension of 10 × 10 mm^2^ with a strip width of 0.5 mm, leading to an effective inductance of 26.7 nH. The conductor loop also had one single gap that was bridged by a thermistor (ERT-J1VA101H, Panasonic, Japan), whose resistance could be easily adjusted by changing the temperature of a heating pad in direct contact with the thermistor. At T=33°C, this thermistor was measured to have 79 Ω resistance, which was approximately equal to the effective impedance of the square inductor at 471.6 MHz (the dipole mode resonance frequency $\omega_{20}$ of the parametric resonator). From $T=25^{\circ }\text{C}$ to 41 °C, the thermistor resistance decreased from 120% to 80% of this optimal resistance (79 Ω). Within this temperature range, the sensor could maintain at least 95% of its maximal frequency response, as defined in (11).

The parametric resonator [Fig. 3(b)] consisted of a 10 × 10 mm^2^ square conductor pattern with two split gaps, both of which were filled by varactor diodes (BBY53-02V, Infineon, Germany) connected in the head-to-head configuration, leading to a dipole mode resonance at $\omega_{20}=471.6\text{MHz}$. By bridging the virtual voltage grounds of the dipole mode with a horizontal conductor in the center, the resonator would have another butterfly mode at 370.2 MHz. When the parametric resonator was overlaid on top of the resistive loop of identical dimension through a 2.6-mm Teflon substrate, the butterfly mode of the parametric resonator was not affected due to geometric orthogonality, but the dipole mode resonance frequency was upshifted. Optionally, another resonant enhancer [Fig. 3(a)] was overlaid on top of the parametric resonator to locally concentrate the magnetic flux at the pumping frequency for improved power efficiency of the pumping field. The resonant enhancer had the same dimension as 10 × 10 mm^2^ but was serially connected to two 2.7-pF chip capacitors, leading to a resonance frequency at 838.3 MHz. When the substrate thickness between the parametric resonator and the resonant enhancer was adjusted to 5.1 mm, the entire circuit assembly would have the highest resonance frequency at 848.0 MHz, which was approximately the sum of the dipole and butterfly resonance frequencies at $\omega_{2}=477.6\;\text{MHz}$ and $\omega_{2b}=370.2\;\text{MHz}$ at 33 °C.

**Fig. 4.**
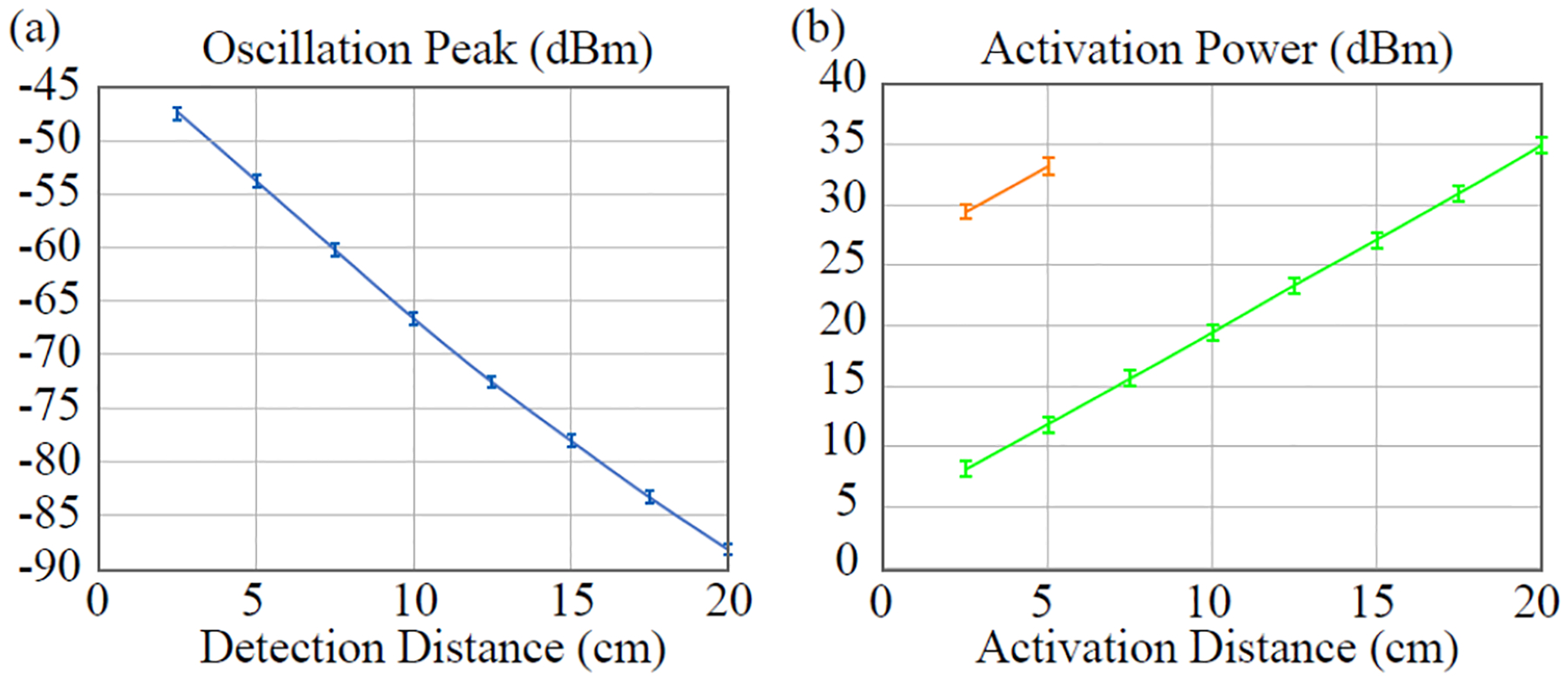
(a) Height of oscillation peak measured under different distance separations between the sensor and the detection antenna. (b) Measured power required on the activation antenna to oscillate the sensor with (green curve) and without (orange curve) the resonant enhancer when the activation antenna was displaced from the sensor by a series of distance separations.

To evaluate the sensor’s remote detectability inside confined cavities, we enclosed the sensor beneath a Petri dish, making its thermistor in direct contact with a heating blanket whose temperature can be adjusted to be within 0.1 °C resolution. The detection antenna was a 9-cm loop that was vertically displaced from the sensor. The activation antenna was a 17.6-cm rod that was horizontally displaced from the sensor by a range of distance separations. The gaps between the sensor and the antennas were filled by several solution-containing flasks to emulate the effect of dissipative tissues with varied thicknesses. The tissue-mimicking solution contained 146% sucrose and 3.6% NaCl, providing similar dielectric constant ($\varepsilon_{r}=50$) and conductivity (σ=0.6S/m) as body tissue [42]. When constant pumping power was applied on the activation antenna, the height of oscillation peak was measured as a function of the distance separation between the sensor and the detection loop. This distance separation was varied by changing the number of the plastic flasks overlaying on top of each other. Each flask had a thickness of 2.5 cm. As shown in Fig. 4(a), every 2.5-cm increase in the detection distance would lead to about a 5.5-dB amplitude decrease of the oscillation peak. Even when the detection antenna was separated by a distance that was 20-fold the sensor’s own dimension, the oscillation peak still had a height of −88 dBm, which was 6 dBm above the noise floor of our spectrum analyzer.

To evaluate the sensor’s activation efficiency, we placed the detection antenna at a location that was about 20-cm above the sensor and varied the distance separation between the activation antenna and the sensor. For each distance separation, the required pumping power was gradually increased until the sensor’s oscillation signal was clearly observable by the detection antenna, showing up as a sharp peak that was 6 dB above the noise floor of the spectrum analyzer. For comparison purpose, the required level of pumping power was also measured on the same sensor but with the resonant enhancer removed. As shown by the orange curve in Fig. 4(b), removal of the resonant enhancer increased the required level of pumping power by ~21 dB. When the antenna was separated from the sensor by more than 7 cm, the sensor could no longer be activated in the absence of resonant enhancer because the required pumping power already exceeded the upper limit of our RF amplifier. On the other hand, by incorporating the resonant enhancer, the sensor could still be activated by ~35 dBm of pumping power even though the activation antenna was displaced from the sensor by 20 cm. This maximum pumping power of 35 dBm was still within the safety level recommended by IEC 60601-2-33, and this distance separation of 20 cm was sufficiently large to reach most deep-lying organs from the torso surface.

## IV. Resistive Sensor Characterization

**Fig. 5.**
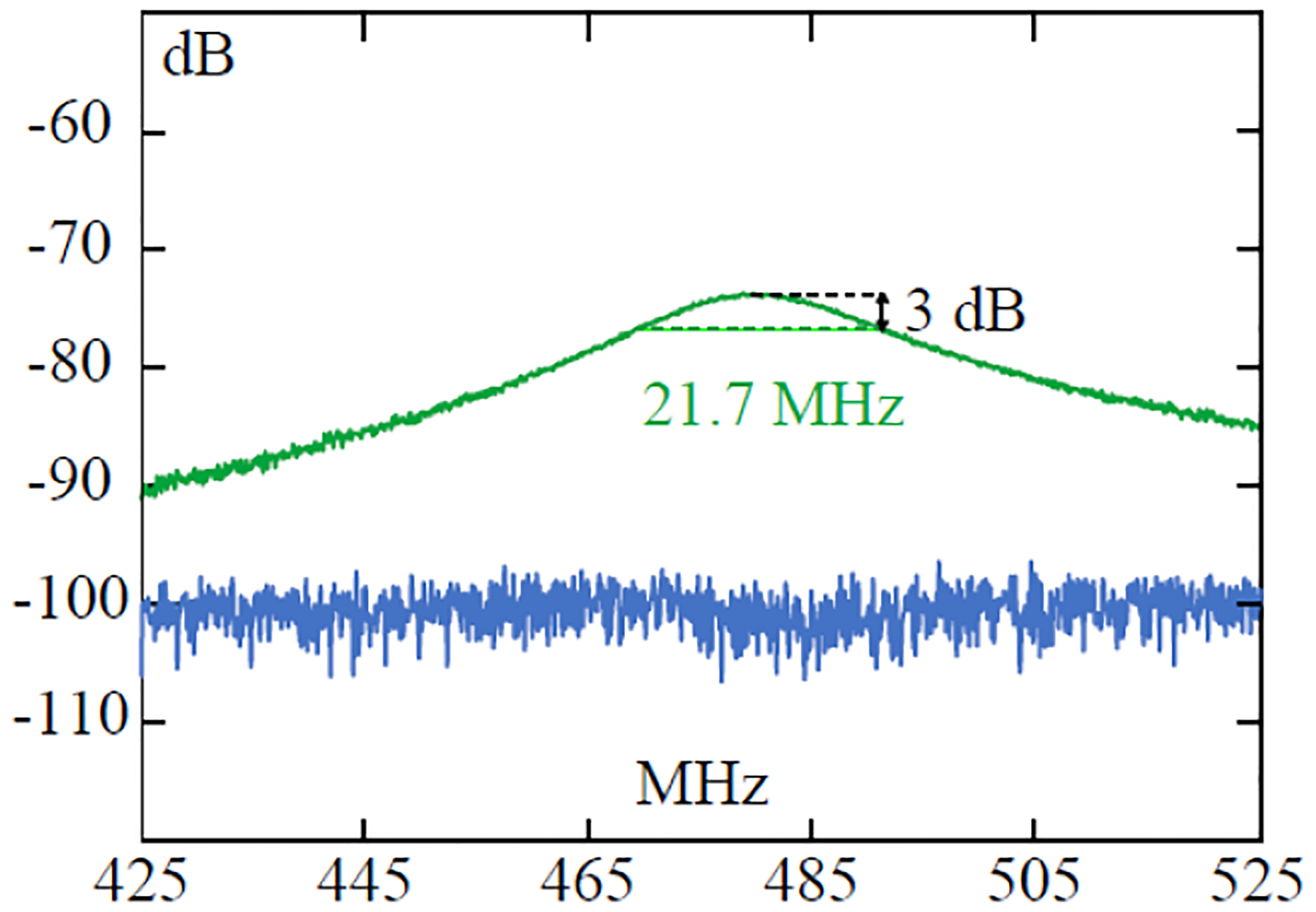
Frequency response curve (green) measured at a 2.5-cm distance separation. When the detection distance was 5 cm, the response curve was completely buried beneath the instrumental noise floor (blue).

Fig. 5 shows the frequency response curve of the resistive sensor directly measured by the backscattering method. This curve was directly measurable when the sensor was within a 2.5-cm distance separation from the detection antenna. However, because the resonator had a 3-dB linewidth of 21.7 MHz, it would be very challenging to precisely measure the much smaller frequency shift induced by 0.1 °C of temperature change. Moreover, when the distance separation was increased to 5 cm, the sensor’s frequency response curve was completely buried beneath the instrumental noise floor (blue), making direct characterization based on the backscattering method even more challenging.

**Fig. 6.**
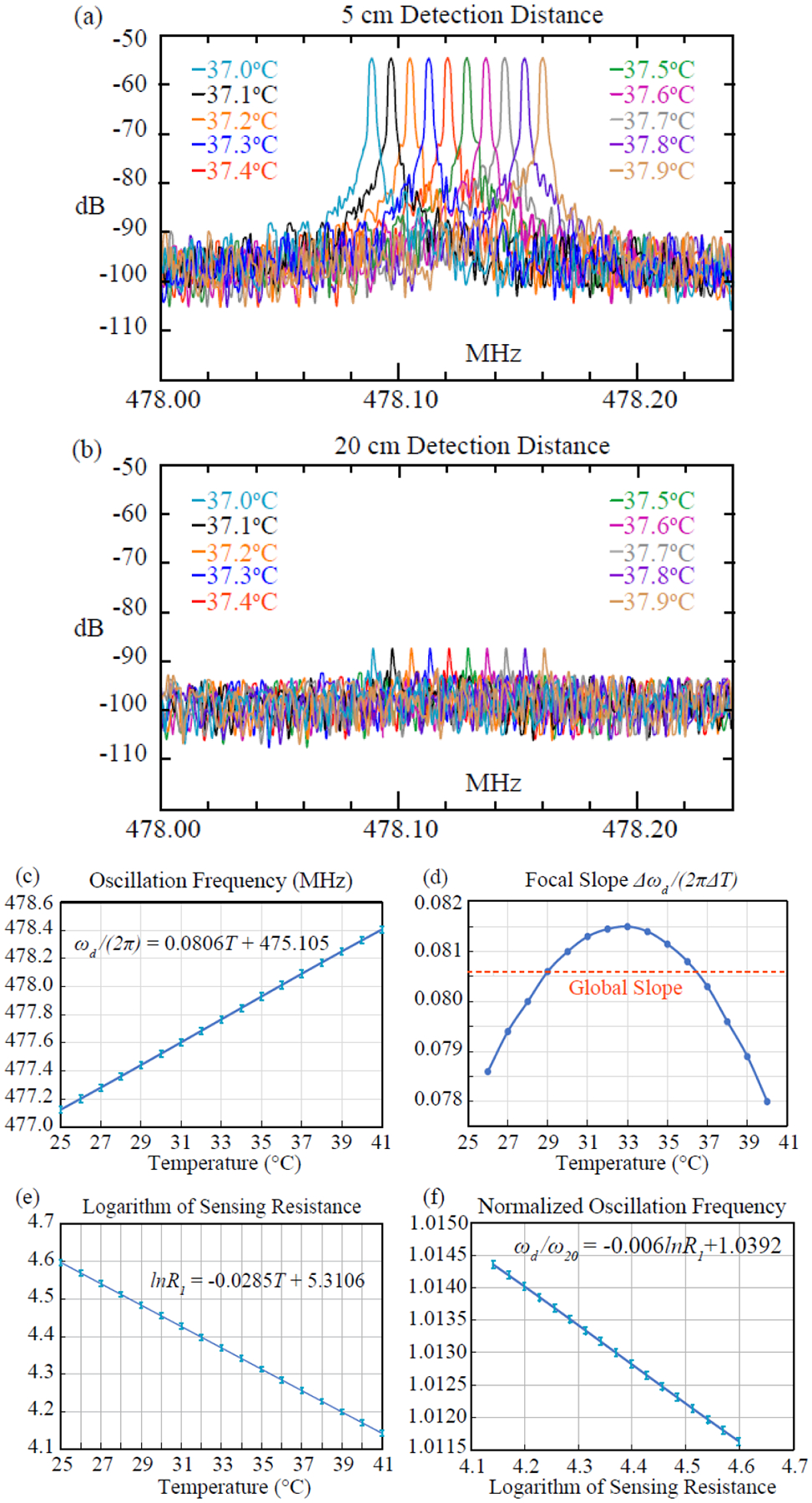
Oscillation signals measured at 0.1 °C temperature intervals when the detection antenna was separated from the sensor by (a) 5 and (b) 20 cm. (c) Linear relation between oscillation frequency and temperature measured at 1 °C interval over a larger range. (d) Focal slopes (solid line) evaluated around individual temperature points are slightly different from the global slope (dashed line) evaluated over the entire temperature range. (e) At each temperature, the thermistor resistance was separately measured, whose logarithm decreased linearly with temperature. (f) Relative change of oscillation frequency $\Delta \omega_{d}/\omega_{20}$ is measured to be proportional to the relative change of resistance $\Delta R_{1}/R_{1}$, as predicted by (10).

At 37 °C, when ~23 dBm of pumping signal at 848.045 MHz was applied on the activation antenna that was separated from the sensor by 12.5 cm, strong oscillation peak [cyan curve in Fig. 6(a)] was observed at 478.087 MHz by the detection antenna that was separated from the sensor by 5 cm. When the temperature of the heating blanket was increased at 0.1 °C interval, the oscillation frequency also shifted linearly. Every 0.1° temperature rise would lead to an 8-kHz increase in oscillation frequency, which was threefold larger than the 3-dB linewidth of each oscillation peak. When we gradually moved the detection antenna away from the resistive sensor, a similar pattern of frequency shift was observed over a range of distance separations. As shown in Fig. 6(b), the oscillation signal was still observable by the detection antenna separated by 20 cm, showing up as sharp peaks that were at least 6 dB larger than the instrumental noise floor. This 20-cm detection distance was already sufficient to reach most deep-lying organs from the body surface.

To quantify the sensor’s frequency response at a 5-cm detection distance, we swept the temperature over a larger range, from 25 °C to 41 °C. At each temperature point, we measured the oscillation frequency five times and calculated its average. As shown in Fig. 6(c), there is a good linear relation between the temperature and the oscillation frequency. The curve’s slope evaluated over the entire temperature range is 0.0806 kHz/° C. However, when focal slopes are evaluated around individual temperature points, they are slightly different from the global average [Fig. 6(d)]. This slight deviation represents the systematic error away from the linear approximation specified in (11). Despite this systematic error, the focal slope evaluated at 37 °C is only ~0.5% different from the average slope, indicating the good accuracy of linear approximation near-physiological temperature.

Besides the apparent relation between temperature and frequency, we also evaluated the underlying relation between resistance variation and oscillation frequency shift. By separately measuring the thermistor’s resistance at each temperature, Fig. 6(e) shows the linear correspondence between every 1 °C temperature rise and every 2.85% resistance decrease (i.e., $-\Delta \left(\text{ln}\;R_{1}\right)=-\Delta R_{1}/R_{1}=2.85\%$). According to Fig. 6(f) that describes the relation between the logarithmic resistance and the normalized oscillation frequency, this $-\Delta R_{1}/R_{1}=2.85\%$ decrease in resistance would lead to $\Delta \omega_{d}/\omega_{20}=171-\text{p}/\text{min}$ increase in oscillation frequency, corresponding to 80.6-kHz increase in oscillation frequency for the parametric resonator with $\omega_{20}/2\pi =471.6\text{MHz}$ in its standalone configuration. This 80.6-kHz frequency increase is consistent with the slope value in Fig. 6(c).

Finally, we repeated the measurement procedure for Fig. 6(c) at multiple detection distances. As shown in Table II, a highly consistent linear relation between the temperature and the oscillation frequency was observed for all distance separations, indicating the reproducibility of the sensor performance.

## V. Discussion

In this work, we have fabricated a compact wireless resistive sensor that can directly convert resistance changes into oscillation frequency shifts for easy detection over large distance separations. This sensor consists of a nonresonant resistive loop that is coaxially overlaid with a parametric resonator. For proof-of-concept demonstration, a thermistor is utilized to provide variable resistance. Through inductive coupling, the resistance change in the resistive loop is converted into reactance change in the parametric resonator. To maximize the sensor’s frequency response, the dimension of the coupling inductor is adjusted to make its reactive impedance $\omega_{20}L_{1}$ approximately equal to the resistance $R_{1}$ of the thermistor at ambient temperature. When $R_{1}$ varies between 80% and 120% of $\omega_{20}L_{1}$ due to temperature variation, there is good linearity between resistance change and oscillation frequency shift of the parametric resonator, maintaining >95% of the sensor’s maximal frequency response. In our prototype demonstration, the coupling inductor $L_{1}$ was fabricated to have reactive impedance of $\omega_{20}L_{1}\sim 79\Omega$, corresponding to a linear range when the sensing resistance $R_{1}$ changes between 63 and 95Ω as a result of temperature variation between 25 ° C and 41 °C. Although this optimal linear range is somewhat narrow, it corresponds to ±20% change in resistance, which is already sufficient for most sensing applications by proper choice of resistive transducers. More importantly, this optimal linear range corresponds to an intermediate regime that was harder to reach by conventional backscattering methods. Previous designs of wireless resistive sensors were based on LC resonators whose quality factors were modulated by a directly connected sensing resistor [29]–[34]. Because the Q-factor was estimated by line shape analysis of the resonator’s frequency response curve, the Q factor had to be reasonably large to ensure sufficient measurement accuracy, necessitating the sensing resistance $R_{1}$ to be either much larger or much smaller than the reactive impedance $\omega_{20}L_{1}$ for parallel or serial connection. In comparison, our inductively coupled parametric resonator can convert resistance changes into linear frequency shifts even when $R_{1}$ is comparable to $\omega_{20}L_{1}$, enabling simplified measurement procedure with better precision, without the need for complicated line shape analysis.

Besides the sensor’s capability for resistance-to-frequency conversion, the parametric resonator can also utilize its non-linear capacitance to convert wireless power provided at the sum frequency into sustained oscillation currents supported by the circuit’s resonance modes. When the applied temperature changes, the resonance frequency of the parametric resonator also shifts due to resistance change in the loop coupler, leading to proportional frequency shift of the resonator’s strong oscillation signal. This self-oscillation feature of the wireless sensor can significantly improve the sensor’s remote detectability, even when the detection distance is as large as 20-fold the sensor’s own dimension. Around body temperature at 37 °C, every 0.1 °C of temperature rise could decrease the effective resistance by 0.285%, leading to 8-kHz increase in oscillation frequency, which is threefold larger than the 3-dB linewidth of the sensor’s oscillation peak. This better than 0.1 °C temperature resolution is sufficient for most applications involving physiological or environmental monitoring.

When the parametric resonator is overlaid with another resonant enhancer that can locally concentrate the magnetic flux at the sum frequency of its resonance modes, the power consumption of the wireless sensor is reduced by ~21 dB. As a result, the wireless sensor can be activated by an external dipole antenna over large distance separations. For example, when the activation antenna is separated from the sensor by 12.5 cm (a distance that is more than enough to reach the center of adult brain), the pumping power required on the activation antenna is only ~23 dBm, making the specific absorption rate well below the safety limit for most regions surrounding the antenna.

Compared to previous designs of wireless resistive sensors, our wireless resistance-to-frequency converter does not require dc voltage to operate, thus obviating the need for extra circuitry and greatly simplifying circuit design. Using a very compact design that requires only a few off-the-shelf components, the wireless resistive sensor is amenable to miniaturize when diodes with larger junction capacitance are utilized to resonate a smaller parametric resonator at similar operation frequency. The sensor’s dimension can be further reduced when thinner substrate layers are utilized to enhance coupling between smaller sized inductors, thus enabling broader range applications. For example, when the wireless sensor is enclosed inside an orthopedic [33] or dental implant [43], it can timely identify abnormal focal temperature changes as early signs of infection. Alternatively, when the wireless oscillator is miniaturized and mounted on the surface of a urinary stent, it can convert the resistance change of piezoresistive transducer [44]–[47] into oscillation frequency shift, enabling chronic monitoring of abnormal renal pressure as an early reminder for stent replacement.

## VI. Conclusion

In summary, we have fabricated a temperature-controlled parametric oscillator that can utilize wireless pumping power provided by an external antenna to directly convert resistance changes of an inductively coupled thermistor into oscillation frequency shifts of the parametric resonator, enabling wireless detection of 0.1 °C temperature change over a 20-cm distance separation (this distance is more than enough to reach most deep-lying organs). Previous designs of wireless sensors were only effective in the near field [29]–[34] unless multiple extra circuit modules were utilized to provide dc power [17]–[22] that was necessary for signal encoding and transmission [14]–[16]. In comparison, our wirelessly powered parametric oscillator has a compact circuit design to integrate power harvesting, signal encoding, and transmission into a single stage, thus greatly improving the remote detectability of resistive transducers for a broad range of physiological and environmental parameters (e.g., humidity, PH, and neuronal voltage) [48]–[50].

## Figures and Tables

**TABLE I:** Summary of Wireless Resistive Sensors

[29–34]	[14]	[15]	[16]	This work
Signal Transmission Mechanism				
Back Scatter	FM Voltage Controlled Oscillator	FSK	ASK	FM Parametric Oscillation
Power Consumption				
Passive	−12 dBm (DC)	−5 dBm (DC)	−17 dBm (DC)	−10 dBm (RF)
Modulated Carrier Frequency				
13.75 MHz	2.3 GHz	433 MHz	150 kHz	478.5 MHz
Temperature Resolution				
1.2°C	0.1°C	0.1°C	0.1°C	0.1°C
Sensor Size				
6.9×4.2 cm^2^	5×5 cm^2^	9×7cm^2^	2.7-cm ring	1×1 cm^2^
Detectable Distance vs. Sensor Size				
~1:1	31:1	~30:1	9:1	20:1
Circuit Complexity				
Low	High	High	High	Low

**TABLE II:** Temperature-Frequency Relation Measured Over Several Distance Separations Between the Sensor and the Detection Antenna

Detection Distance	Oscillation Freq (MHz)
5 cm	0.0806T+475.105
10 cm	0.0804T+475.112
15 cm	0.0807T+475.101
20 cm	0.0805T+475.108

## Appendix

In (9), the first line describes the approximate linear relation between the oscillation frequency $\omega_{d}$ and the resonance frequency $\omega_{2}$. In the following, we will prove that the second line of (9) is negligible:

(12)
$$\frac{\left(\omega_{p}-\omega_{2b}-\omega_{2}\right)}{\omega_{20}}R_{1}\frac{d}{dR_{1}}\left(\frac{L_{2b}/R_{2b}}{L_{2b}/R_{2b}+L_{2d}/R_{2d}}\right)\;=\frac{\left(\omega_{p}-\omega_{2b}-\omega_{2}\right)}{\omega_{20}}\left(\frac{R_{1}}{R_{2d}}\right)\left(\frac{L_{2d}L_{2b}R_{2b}R_{2d}}{{\left(L_{2b}R_{2d}+L_{2d}R_{2b}\right)}^{2}}\right)\frac{dR_{2d}}{dR_{1}}\;=\frac{\left(\omega_{p}-\omega_{2b}-\omega_{2}\right)}{\omega_{20}}\frac{1}{{\left(\sqrt{\frac{L_{2d}R_{2b}}{L_{2b}R_{2d}}}+\sqrt{\frac{L_{2b}R_{2d}}{L_{2d}R_{2b}}}\right)}^{2}}\left(\frac{R_{1}dR_{2d}}{R_{2d}dR_{1}}\right)\;\leq \left(\frac{\omega_{p}-\omega_{2b}-\omega_{2}}{4\omega_{20}}\right)\left(\frac{R_{1}dR_{2d}}{R_{2d}dR_{1}}\right).$$

In (12), the factor $\left(\omega_{p}-\omega_{2b}-\omega_{2}\right)/\omega_{20}$ is zero when $\omega_{p}$ is set to $\omega_{2b}+\omega_{2}$ for a specific $R_{1}$. When $R_{1}$ changes by a small portion around its original value, only $\omega_{2}$ responds to this change. By plugging in (6), the first term in the last line of (12) satisfies the following relation:

(13)
$$\left|\frac{\omega_{p}-\omega_{2b}-\omega_{2}}{4\omega_{20}}\right|\;=\left|\frac{\Delta \omega_{2}}{4\omega_{20}}\right|\approx \frac{\kappa^{2}\left|\Delta R_{1}/R_{1}\right|}{4\left(1-\kappa^{2}\right){\left(\frac{R_{1}}{L_{1}\omega_{20}}+\frac{L_{1}\omega_{20}}{R_{1}}\right)}^{2}}.$$

On the other hand, the effective resistance $R_{2d}$ in (1) is

(14)
$$R_{2d}=\frac{\omega_{2}^{2}k^{2}L_{1}L_{2}}{R_{1}+\omega_{2}^{2}L_{1}^{2}/R_{1}}+R_{2}\;=\frac{k^{2}}{\left(\frac{R_{1}}{\omega_{20}L_{1}}\right)\left(\frac{\omega_{20}}{\omega_{2}^{2}L_{2}}\right)+\left(\frac{\omega_{20}L_{1}}{R_{1}}\right)\left(\frac{1}{\omega_{20}L_{2}}\right)}+R_{2}\;=\frac{k^{2}}{\left(\frac{R_{1}}{\omega_{20}L_{1}}\right)\left(\frac{1}{\omega_{20}L_{2}}\right)\left(\frac{\omega_{20}^{2}}{\omega_{2}^{2}}\right)+\left(\frac{\omega_{20}L_{1}}{R_{1}}\right)\left(\frac{1}{\omega_{20}L_{2}}\right)}+R_{2}.$$

By plugging (5) into (14)

(15)
$$\frac{R_{2d}}{\omega_{20}L_{2}}=\frac{k^{2}}{\left(\frac{R_{1}}{\omega_{20}L_{1}}\right)/\left(1+\frac{\kappa^{2}}{\left(1+R_{1}^{2}/\left(\omega_{20}^{2}L_{1}^{2}\right)\right)\left(1-\kappa^{2}\right)}\right)+\left(\frac{\omega_{20}L_{1}}{R_{1}}\right)}+\frac{R_{2}}{\omega_{20}L_{2}}.$$

By defining the normalized resistance as $Rr\equiv R_{1}/\omega_{20}L_{1}$, the last factor $\left(R_{1}dR_{2d}\right)/\left(R_{2d}dR_{1}\right)$ in (12) can be estimated by taking derivative on both sides of (15)

(16)
$$\left(\frac{R_{1}}{R_{2d}}\right)\frac{d\left(R_{2d}\right)}{d\left(R_{1}\right)}\;=\left(\frac{R_{1}}{R_{2d}}\right)\frac{d\left(R_{2d}/\omega_{20}L_{2}\right)}{d\left(R_{1}/\omega_{20}L_{1}\right)}\left(\frac{L_{2}}{L_{1}}\right)\;=\left(\frac{R_{1}L_{2}\omega_{20}k^{2}}{L_{1}\omega_{20}R_{2d}}\right)\;\times \frac{\left(1+\left(1-k^{2}\right)Rr^{2}-\left(1+k^{2}-2k^{4}\right)Rr^{4}-{\left(1-k^{2}\right)}^{2}Rr^{6}\right)}{{\left(k^{2}+\left(1-k^{2}\right){\left(1+Rr^{2}\right)}^{2}\right)}^{2}}\;=\left(\frac{k^{2}L_{2}\omega_{20}}{R_{2d}}\right)\;\times \frac{Rr\left(1+\left(1-k^{2}\right)Rr^{2}-\left(1+k^{2}-2k^{4}\right)Rr^{4}-{\left(1-k^{2}\right)}^{2}Rr^{6}\right)}{{\left(k^{2}+\left(1-k^{2}\right){\left(1+Rr^{2}\right)}^{2}\right)}^{2}}\;\approx \left(\frac{k^{2}L_{2}\omega_{20}}{R_{2d}}\right)\frac{\left(Rr-Rr^{3}\right)}{{\left(1+Rr^{2}\right)}^{2}}.$$

The last approximation holds when the inductive coupling coefficient $k^{2}\ll 1$. By plugging (15) into (16)

(17)
$$\frac{R_{1}d\left(R_{2d}\right)}{R_{2d}d\left(R_{1}\right)}\;\approx \left(\frac{k^{2}}{\frac{k^{2}}{Rr/\left(1+\frac{\kappa^{2}}{\left(1+Rr^{2}\right)\left(1-k^{2}\right)}\right)+\left(\frac{1}{Rr}\right)}+\frac{R_{2}}{\omega_{20}L_{2}}}\right)\frac{\left(Rr-Rr^{3}\right)}{{\left(1+Rr^{2}\right)}^{2}}\;<\left(\text{Rr}/\left(1+\frac{\kappa^{2}}{\left(1+Rr^{2}\right)\left(1-\kappa^{2}\right)}\right)+\left(\frac{1}{Rr}\right)\right)\frac{\left(Rr-Rr^{3}\right)}{{\left(1+Rr^{2}\right)}^{2}}\;<\left(Rr+\frac{1}{Rr}\right)\frac{\left(Rr-Rr^{3}\right)}{{\left(1+Rr^{2}\right)}^{2}}=\frac{1-Rr^{2}}{1+Rr^{2}}\text{.}$$

When Rr=1, (17) is 0, indicating that $R_{2d}$ remains almost constant when $R_{1}$ changes around its optimal value at $\omega_{20}L_{1}$. When Rr varies from 0.8 to 1.2, (6) decreases from 0.22 to −0.18, thus defining the following boundary condition:

(18)
$$\left|\frac{R_{1}d\left(R_{2d}\right)}{R_{2d}d\left(R_{1}\right)}\right|<\frac{1}{4.5}.$$

By plugging (13) and (18) into (12)

(19)
$$\left|\frac{\left(\omega_{p}-\omega_{2b}-\omega_{2}\right)}{4\omega_{20}}\right|\left|\frac{R_{1}dR_{2d}}{R_{2d}dR_{1}}\right|<\frac{\kappa^{2}}{\left(1-\kappa^{2}\right){\left(\frac{R_{1}}{L_{1}\omega_{20}}+\frac{L_{1}\omega_{20}}{R_{1}}\right)}^{2}}\left(\frac{1}{4\times 4.5}\right)\left|\frac{\Delta R_{1}}{R_{1}}\right|.$$

Meanwhile, the first line of (9) can be estimated as

(20)
$$\left|\frac{d\left(\omega_{2}/\omega_{20}\right)}{dR_{1}/R_{1}}\left(1-\frac{L_{2b}/R_{2b}}{L_{2b}/R_{2b}+L_{2}/R_{2d}}\right)\right|\;=\frac{\kappa^{2}}{\left(1-\kappa^{2}\right){\left(\frac{R_{1}}{L_{1}\omega_{20}}+\frac{L_{1}\omega_{20}}{R_{1}}\right)}^{2}}\left(1-\frac{L_{2b}/R_{2b}}{L_{2b}/R_{2b}+L_{2}/R_{2d}}\right)\;\approx \frac{\kappa^{2}}{\left(1-\kappa^{2}\right){\left(\frac{R_{1}}{L_{1}\omega_{20}}+\frac{L_{1}\omega_{20}}{R_{1}}\right)}^{2}}\left(1-\frac{L_{2b}/R_{2b}}{L_{2b}/R_{2b}+L_{2}/\left(2R_{2}\right)}\right)\;\approx \frac{\kappa^{2}}{\left(1-\kappa^{2}\right){\left(\frac{R_{1}}{L_{1}\omega_{20}}+\frac{L_{1}\omega_{20}}{R_{1}}\right)}^{2}}\left(\frac{1}{3}\right).$$

The first approximation in (20) is made under the design feature that the resistive loop can increase the effective resistance of the parametric resonator from $R_{2}$ to $R_{2d}\sim 2R_{2}$. The second approximation in (20) is made under the approximation that when the parametric resonator is in its stand-alone configuration, its dipole and butterfly modes have a similar inductance-to-resistance ratio, i.e., $L_{2b}/R_{2b}=L_{2}/R_{2}$.

When comparing (19) with (20), the former is at most 1/30 of the latter when $\left|\Delta R_{1}/R_{1}\right|$ reaches its boundary at 0.2. As a result, only the first line in (9) is maintained to specify the linear relation between the relative change in oscillation frequency $d\omega_{d}/\omega_{20}$ and the relative change in sensing resistance $dR_{1}/R_{1}$.
